# Breast Cancer Stem-Like Cells Are Inhibited by a Non-Toxic Aryl Hydrocarbon Receptor Agonist

**DOI:** 10.1371/journal.pone.0013831

**Published:** 2010-11-03

**Authors:** Gérald J. Prud'homme, Yelena Glinka, Anna Toulina, Olga Ace, Venkateswaran Subramaniam, Serge Jothy

**Affiliations:** 1 Department of Laboratory Medicine and Li Ka Shing Knowledge Institute, St Michael's Hospital, Toronto, Canada; 2 Department of Laboratory Medicine and Pathobiology, University of Toronto, Toronto, Canada; University of Minnesota, United States of America

## Abstract

**Background:**

Cancer stem cells (CSCs) have increased resistance to cancer chemotherapy. They can be enriched as drug-surviving CSCs (D-CSCs) by growth with chemotherapeutic drugs, and/or by sorting of cells expressing CSC markers such as aldehyde dehydrogenase-1 (ALDH). CSCs form colonies in agar, mammospheres in low-adherence cultures, and tumors following xenotransplantation in Scid mice. We hypothesized that tranilast, a non-toxic orally active drug with anti-cancer activities, would inhibit breast CSCs.

**Methodology/Findings:**

We examined breast cancer cell lines or D-CSCs generated by growth of these cells with mitoxantrone. Tranilast inhibited colony formation, mammosphere formation and stem cell marker expression. Mitoxantrone-selected cells were enriched for CSCs expressing stem cell markers ALDH, c-kit, Oct-4, and ABCG2, and efficient at forming mammospheres. Tranilast markedly inhibited mammosphere formation by D-CSCs and dissociated formed mammospheres, at pharmacologically relevant concentrations. It was effective against D-CSCs of both HER-2+ and triple-negative cell lines. Tranilast was also effective *in vivo*, since it prevented lung metastasis in mice injected *i.v.* with triple-negative (MDA-MB-231) mitoxantrone-selected cells. The molecular targets of tranilast in cancer have been unknown, but here we demonstrate it is an aryl hydrocarbon receptor (AHR) agonist and this plays a key role. AHR is a transcription factor activated by 2,3,7,8-tetrachlorodibenzo-*p*-dioxin (TCDD), polycyclic aromatic hydrocarbons and other ligands. Tranilast induced translocation of the AHR to the nucleus and stimulated CYP1A1 expression (a marker of AHR activation). It inhibited binding of the AHR to CDK4, which has been linked to cell-cycle arrest. D-CSCs expressed higher levels of the AHR than other cells. Knockdown of the AHR with siRNA, or blockade with an AHR antagonist, entirely abrogated the anti-proliferative and anti-mammosphere activity of tranilast. Thus, the anti-cancer effects of tranilast are AHR dependent.

**Conclusion/Significance:**

We show that tranilast is an AHR agonist with inhibitory effects on breast CSCs. It is effective against CSCs of triple-negative breast cancer cells selected for anti-cancer drug resistance. These results suggest it might find applications in the treatment of breast cancer.

## Introduction

Cancer stem cells (CSCs), also denoted cancer initiating cells (CIC), have increased resistance to cancer chemotherapy, and there is an urgent need to identify drugs that target these cells. They were first identified in human leukemias [1], and more recently in solid tumors [2], [3]. A minimal definition of CSCs has three features [2]–[4]: 1) Distinct markers allowing purification; 2) highly tumorigenic as compared to other subsets; and 3) ability to differentiate to recreate all cell phenotypes of the parent tumor. There are several other notable features such as self-renewal, the capacity to form tumor spheres in low-adherence cultures, expression of high levels of ATP-binding cassette (ABC) drug transporters (especially ABCG2) and multi-drug resistance. Human breast cancer cell lines (e.g., MDA-MB-231, BT474 and SUM159) can be fractionated into subpopulations, some of which have CSC properties and form mammospheres in vitro. CSCs can be isolated by fluorescence-activated cell sorting (FACS) within the Hoescht 33342 dye-excluding side population (SP) [5], or the aldehyde dehydrogenase-1 high (ALDH^hi^) population [6], [7]. Importantly, recent studies [8]–[10] have shown that CSC-like cells can also be enriched from cancer cell lines by growth with conventional chemotherapeutic drugs (e.g., doxorubicin, cisplatin, etoposide, mitoxantrone). In this study, we generated drug-surviving CSCs (D-CSCs) by growing breast cancer cells in mitoxantrone. The growth of breast cancer cell lines in medium containing mitoxantrone resulted in a marked enrichment of cells with CSC-like markers (ALDH^hi^, c-kit, Oct-4 and ABCG2) and functional characteristics. This method has the advantage of selecting the cells for a property (drug resistance) that is highly relevant to cancer therapy.

We hypothesized that tranilast, a drug with anti-proliferative and anti-cancer activities [11]–[13], would inhibit CSCs. It is a non-toxic orally active drug developed for allergic diseases [14], but that we found targets breast cancer cells through multiple pathways [12], [13]. Tranilast inhibits the following: cell cycling, TGF-β activity, MAPK signaling, epithelial-to-mesenchymal transition (EMT), cell migration and invasion. In vivo, it has prominent anti-metastatic effects [12]. Here we show that tranilast strongly inhibits CSCs in colony forming assays and mammosphere formation assays, at pharmacologically relevant concentrations. Tranilast was highly active at inhibiting mammosphere formation by D-CSCs, and dissociating formed mammospheres. It was effective in vivo, preventing metastasis to the lungs following i.v. injection of MDA-MB-231 mitoxantrone-selected cells.

The molecular target(s) of tranilast in cancer have been unclear, but we demonstrate here that it is an aryl hydrocarbon receptor (AHR) agonist. The AHR is a transcription factor known principally as a receptor for toxins such as 2,3,7,8-tetrachlorodibenzo-*p*-dioxin (TCDD) [15], but it has other functions [16] and appears to exert some anti-cancer effects [17], [18]. We show that tranilast binds to the AHR, induces its translocation to the nucleus, and stimulates CYP1A1 expression (a classic marker of AHR activity). D-CSCs expressed increased amounts of AHR. Knockdown of the AHR with siRNA, or addition of an AHR antagonist to cultures, both abolished the activity of tranilast in vitro. Thus, the activity of tranilast appears to be highly AHR dependent.

We also show that tranilast is effective at inhibiting CSCs of triple-negative (ER−/PR−/HER-2−) breast cancer cell lines (MDA-MB-231, SUM149 and SUM159) or a triple-positive cell line (ER+/PR+/HER-2+) (BT474). This is particularly relevant because triple-negative cells have lacked a distinct molecular target and their treatment is problematic. Recent studies suggest that the AHR is an excellent target for breast cancer therapy [17], [18], and tranilast appears to be an exceptional drug for this purpose.

## Results

### Inhibition of colony formation and mammosphere formation by tranilast

**Figure 1:**
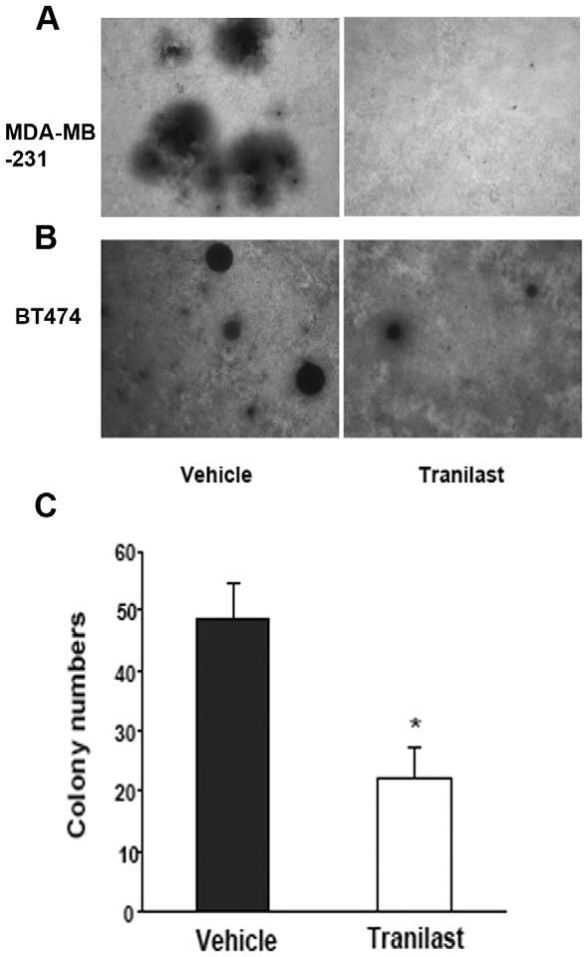
Tranilast treatment reduces colony size and numbers. A and B. Colony forming assay using soft agar of MDA-MB-231 cells (A) or BT474 cells (B). In both cases we observed a reduction in colony size in tranilast treated cells when compared to the vehicle control (day 12, 400x). C. The colony numbers of BT474 were significantly reduced (mean + SD, *p<0.05). Reduction in colony numbers with MDA-MB-231 cells was equally significant (not shown). Results shown are the mean of 2 independent experiments in triplicates.

One property of CSCs is the ability to form colonies in soft agar. When tranilast was added to cultures of MDA-MB-231 or BT474 breast cancer cells at 200 µM there was marked inhibition of colony formation (Fig. 1A–C). The colonies were significantly fewer and smaller. Note that the MDA-MB-231 colonies, compared to BT474, are not as round and their borders are less clearly defined. We have found this to be a regular feature of MDA-MB-231 colonies.

**Figure 2:**
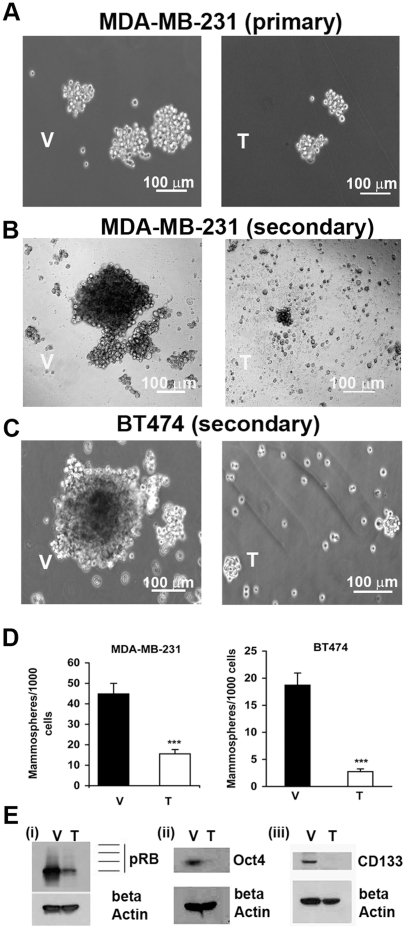
Tranilast treatment reduces mammosphere formation and stem cell marker expression. A. Tranilast (200 µM) markedly reduced the number of primary mammospheres of MDA-MB-231 cell line when compared to the vehicle control (results at d 7) (100X). B. Similar results were obtained in MDA-MB-231 secondary mammosphere culture. C. Tranilast (200 µM) markedly reduced the numbers and size of secondary mammospheres (results at d 14) of the BT474 cell line (100X). D. Tranilast caused a significant reduction of mammosphere numbers related to the number of cells plated as described in Fig. 2A and 2C (mean + SD; ***p<0.0001). Results are the mean of 2 independent experiments in triplicates. The mean diameter of the mammospheres in tranilast cultures was significantly less than half that of vehicle cultures (not shown). Scale bar: 100 µm. E,i. Western blots of MDA-MB-231 cell lysates of primary mammospheres show that phosphorylated retinoblastoma protein (denoted here pRB) was greatly reduced by tranilast. E, ii, iii. Western blots show complete suppression of the stem cell markers Oct 4 and CD133 by tranilast. The data presented in Fig. 2A–E were all derived from cells grown under mammosphere culture conditions with 200 µM tranilast or vehicle. V  =  vehicle; T  =  tranilast.

Another property of CSCs is the ability to form tumor spheres, denoted mammospheres in the case of breast cancer. Unselected breast cancer cells (parental population) of different phenotype [19], [20] were plated in serum-free, low adherence cultures to generate mammospheres. Cells derived from the MDA-MB-231 cell line (ER^−^/PR^−^/HER-2^−^ triple-negative cell line) (Fig. 2A,B), or the BT474 cell line (ER^+^/PR^+^/HER-2^+^ triple-positive) (Fig. 2C), both readily formed mammospheres in this assay. The shape and appearance of the spheres was cell-line dependent, and BT474 cells tended to form round spheres, while MDA-MB-231 mammospheres were looser and less rounded. Tranilast markedly inhibited the formation of mammospheres in primary and secondary mammosphere assays of these cells lines (Fig. 2A–D).

Furthermore, Western blotting studies revealed that tranilast suppressed the phosphorylation of retinoblastoma protein (RB), and the expression of CSC markers CD133 and Oct-4 in MDA-MB-231 mammosphere cultures (Fig. 2E).

To determine whether tranilast eliminated sphere-forming CSCs, we isolated cells grown with tranilast, as in Fig. 2A, and replated the cells in mammosphere cultures without tranilast (Fig. S1A). The replated cells formed mammospheres, indicating that at least a portion of sphere forming cells survived incubation with tranilast. This is consistent with our previous findings that tranilast is not markedly cytotoxic [12]. Nevertheless, tranilast reduced cell survival (Fig. S1B) in mammosphere culture, as discussed below.

### Isolation of D-CSCs

**Figure 3:**
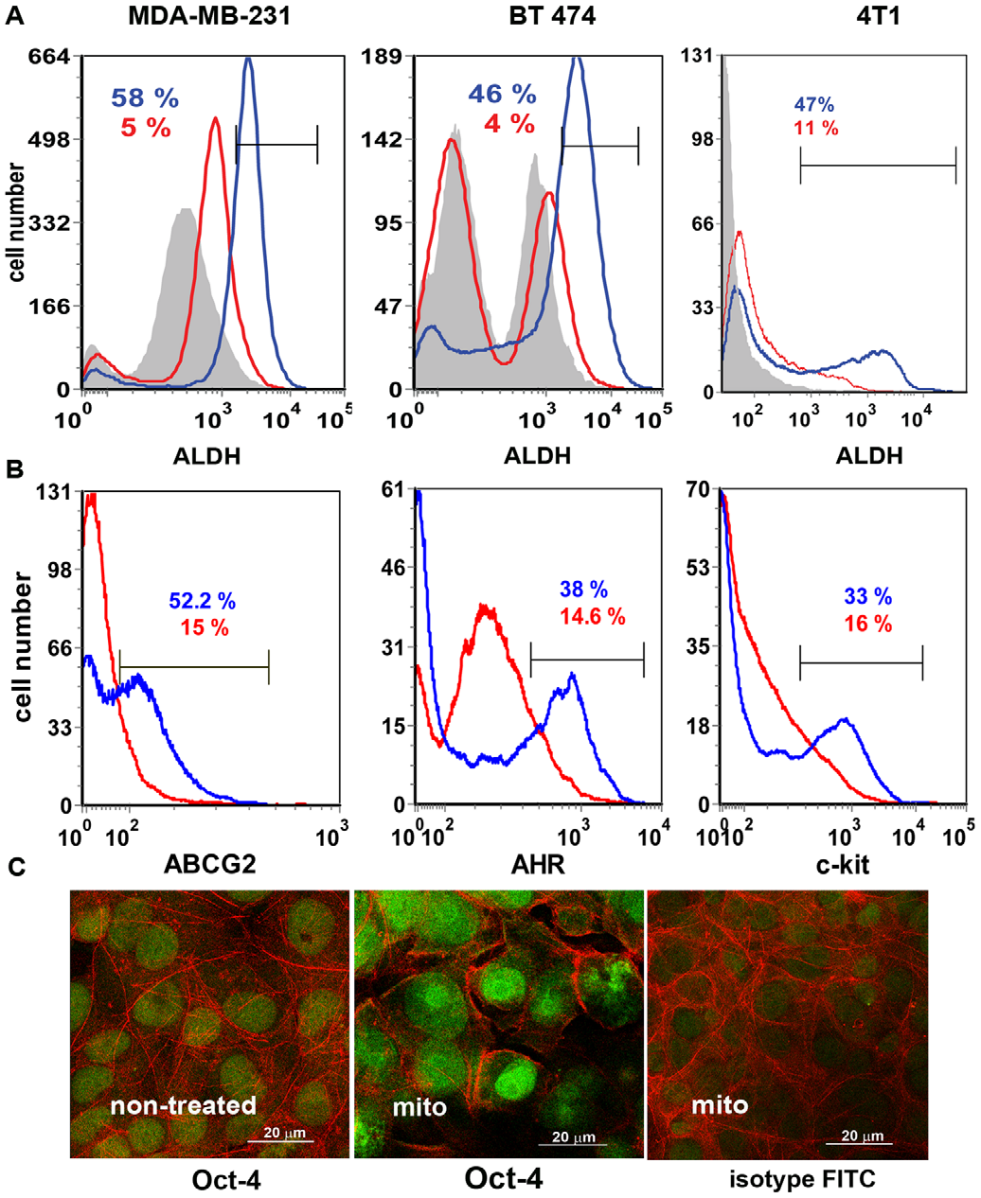
Drug-selected cells express higher level of stem cell markers. Phenotyping of mitoxantrone-selected cells. A. Cells from cell lines MDA-MB-231, BT474, or 4T1 were grown in mitoxantrone-containing medium to generate CSC-like cells (D-CSCs), as described in Materials and Methods. The cells were stained for ALDH-1 expression by the ALDEFLUOR assay. Silver-filled histograms represent negative control obtained in the presence of the ALDH-1 inhibitor DEAB, and were used for the gating of the ALDH-1 bright cells (ALDH high). In each histogram, blue lines and numbers (%) represent the percent of ALDH high cells in mitoxantrone-treated cells, while red lines and numbers (%) represent the percent of ALDH high cells in the non-treated cells. The figures show that mitoxantrone notably increased the number of ALDH1 high cells. B. MDA-MB-231 cells were stained for ABCG2, AHR, or c-kit and analyzed by flow cytometry. Mitoxantrone-selected cells (blue lines and numbers [%]) showed a marked increased in the expression of ABCG2, AHR, and c-kit, as compared to the non-selected cells (red lines and numbers [%]). The gates were positioned to include the peak of the brightest cells in each histogram. C. Drug selection increased the number of cells expressing the stem cell nuclear marker Oct-4 (green) in MDA-MB-231 cells, from ∼5% without mitoxantrone (non-treated) to ∼30% with mitoxantrone (mito), as detected by immunofluorescent confocal microscopy. The cytoplasm (red) was decorated with Alexa Fluor 568-conjugated phalloidin that binds to actin. The data in panels A and B are representative of two independent experiments.

There are no completely specific markers of breast CSCs, but the cells can be enriched by sorting for some markers such as CD44+/CD24-(or low)/ESA+ [19] or ALDH^hi^ expression (ALDEFLUOR reaction) [6], [7]. However, there is limited correlation in the expression of these markers in different studies, and some cell lines such as MDA-MB-231 consist mainly of CD44^+^/CD24^−^ cells. An alternative approach to enriching CSCs involves the isolation of drug-surviving CSC-like cells, that we denote D-CSCs, as described by Levina et al. [8] and others [9], [10]. In this case, the cells which survive culture with doxorubicin, mitoxantrone or other drugs are highly enriched in cells with CSC markers and functional features. In this study, we produced D-CSCs by growing breast cancer cell lines, such as MDA-MB-231 and BT474, in mitoxantrone-containing medium. After 5 days of mitoxantrone exposure, the cells were transferred to mitoxantrone-free culture medium for another 3–5 days before further studies were performed. As detected by flow cytometric analysis (Fig. 3A,B) or confocal microscopy (Fig. 3C) the mitoxantrone-selected cells were markedly enriched for CSC marker expression, including ALDH^hi^, c-kit, ABCG2 and Oct-4. There was also increased expression of the AHR (Fig. 3B). Hence, although the AHR is expressed by the parental cells and is not a specific CSC marker, its expression was markedly increased by mitoxantrone (see below).

Thus, the drug-selected cells were markedly enriched in cells with CSC markers, with 30–50% of the cells expressing at least one of these markers. The high level of expression of ABCG2 is consistent with the ability of this drug transporter to pump mitoxantrone out of the cells [21], [22]. In addition, by qRT-PCR array analysis (SABiosciences), the D-CSCs overexpress (>2-fold) several stem-cell related genes compared to the parental line (e.g., ABCG2, ALDH-1, BMPs, FGFs, desert hedgehog, WNT1) (data not shown). The drug-surviving cells also formed mammospheres at higher frequency as detailed below.

### Activity of tranilast against mammosphere-forming D-CSCs

**Figure 4:**
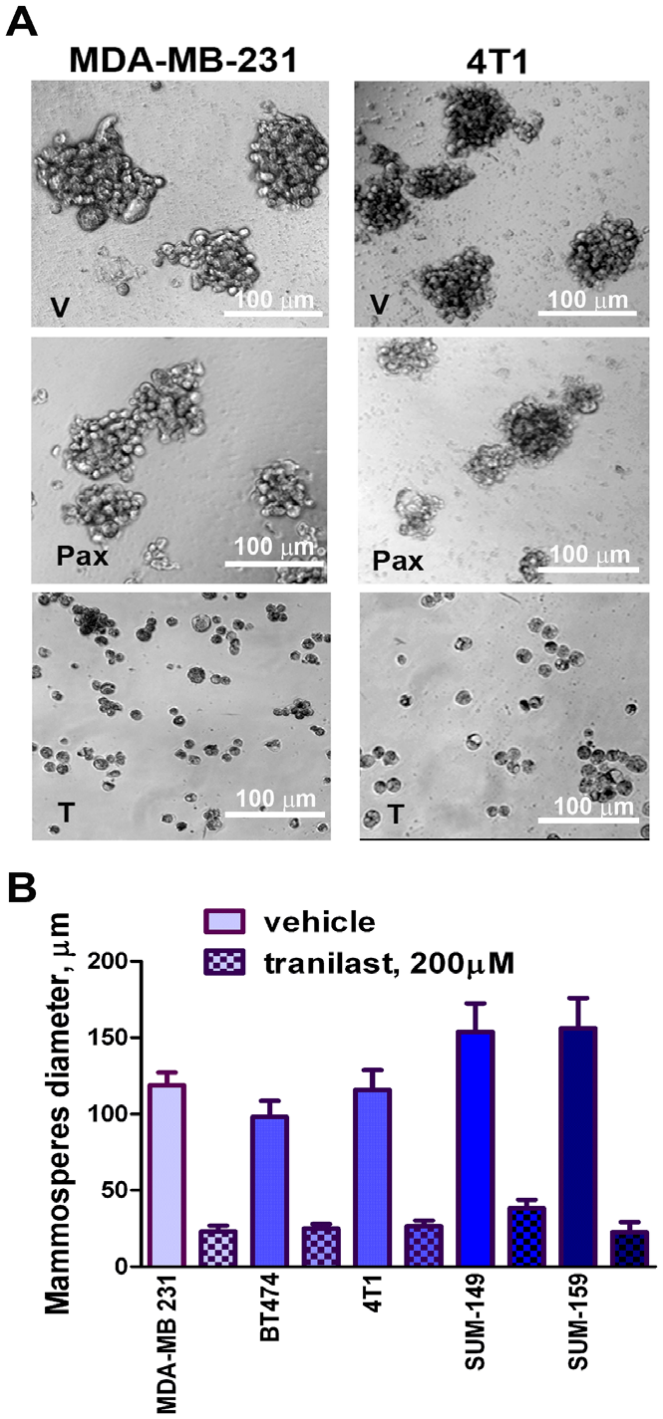
Dissociation of mammospheres after treatment with tranilast. A. MDA-MB-231 and 4T1 cells were selected by growth with mitoxantrone. The live cells (after a period of growth in mitoxantrone-free medium) were recovered and then grown in mammosphere culture. After 7 days mammospheres had formed at high frequency, and at that point vehicle (V, 1st row; DMSO), paclitaxel (Pax, 2nd row; 20 nM) or tranilast (T, 3rd row; 200 µM) were added to the cultures. After a further 48 hours in culture mammospheres were reduced in size but not disrupted in the paclitaxel cultures, whereas they were completely dissociated in the tranilast cultures. Similar mammosphere disruption occurred at tranilast concentrations of 100 and 400 µM (not shown.). B. Tranilast was added to formed mammospheres of different breast cancer cell lines, as described above for Fig. 4A. Tranilast was effective at dissociating mammospheres of all breast cancer cell lines tested. MDA-MB-231, SUM149 and SUM159 (all human), and 4T1 (mouse), are all ER-/PR-/HER-2- (triple-negative) cell lines. BT474 is an ER+/PR+/HER-2+ (triple-positive) human cell line. The results represent the mean diameter of mammospheres +/− SEM, with or without tranilast. In panels A and B, three experiments yielded similar results.

Previously [12], we found that a rat mammary carcinoma line with stem cell features (LA7) was highly sensitive to tranilast. Furthermore, tranilast inhibited EMT [12], which has been linked to the CSC phenotype [23], [24]. Mammosphere culture has been shown to enrich for tumorigenic breast cancer cells [25]. Here, we show that when tranilast was added to mammospheres that were already formed, generated from mitoxantrone-selected MDA-MB-231 (human) or 4T1 (mouse) cells, the spheres dissociated within 48 h (Fig. 4A). Tranilast also dissociated mammospheres produced by other breast cancer cell lines including BT474, SUM149 and SUM159 (Fig. 4B). Dissociation of the tranilast-treated spheres was associated with increased cell death, but ≥50% of cells were still alive after dissociation (trypan blue dye exclusion assay; not shown), suggesting that it was not due to cell death alone. The dissolution of existing spheres is an important finding, because it suggests that the anti-mammosphere effect of tranilast is not solely due to an inhibition of proliferation.

**Figure 5:**
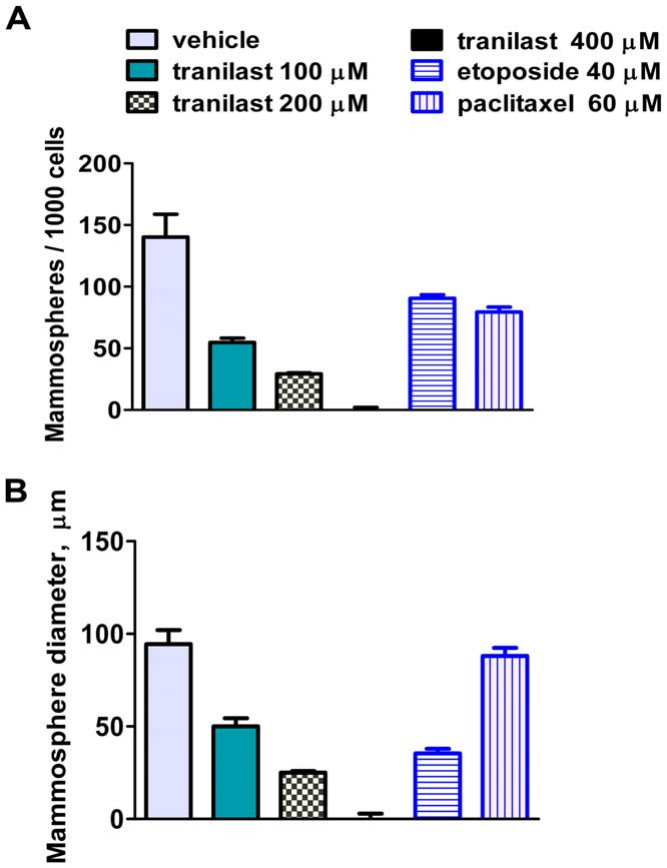
Tranilast inhibits mammosphere formation by mitoxantrone-selected MDA-MB-231 cells. MDA-MB-231 mitoxantrone-selected cells were plated for mammosphere formation (1000 cells/well; 96-well plate, Cnt-27 medium) and grown with tranilast (100–400 µM), or a high concentration of either etoposide (40 µM) or paclitaxel (60 µM). Control cells were grown with vehicle (2.5% DMSO). Tranilast decreased the numbers (A) and size (B) of the mammospheres, such that none were visible at 400 µM. Tranilast appeared more effective than etoposide or paclitaxel at reducing mammosphere numbers (mean + SEM; p<0.05 for tranilast at 200 µM versus either vehicle, etoposide or paclitaxel). The data are representative of two independent experiments.

Mitoxantrone-selected cells formed mammospheres at a higher frequency (Fig. 5) than unselected cells (Fig. 2D). Tranilast inhibited formation of mammospheres from mitoxantrone-selected MDA-MB-231 cells when it was added at the start of cultures (Fig. 5A,B). Inhibition occurred at tranilast concentrations ≥100 µM, and at 400 µM no mammospheres were seen. Tranilast reduced cell survival in these cultures, but even at the highest concentration (400 µM) almost 50% of cells survived after 7 d in culture (Fig. S1B). Note that at concentrations that can be achieved pharmacologically (100–200 µM) some spheres still formed (although they were smaller), suggesting survival of sphere-forming cells.

We compared the effects of tranilast with paclitaxel and etoposide at various concentrations. In Fig. 5A,B, the results obtained with the highest concentrations of either paclitaxel (60 µM) or etoposide (40 µM) tested are shown. Lower concentrations were less effective (not shown). As seen in Fig. 5A, pharmacological concentrations of tranilast (100–200 µM) were more effective than these drugs at reducing the number of mammospheres.

### Tranilast is an AHR agonist

**Figure 6:**
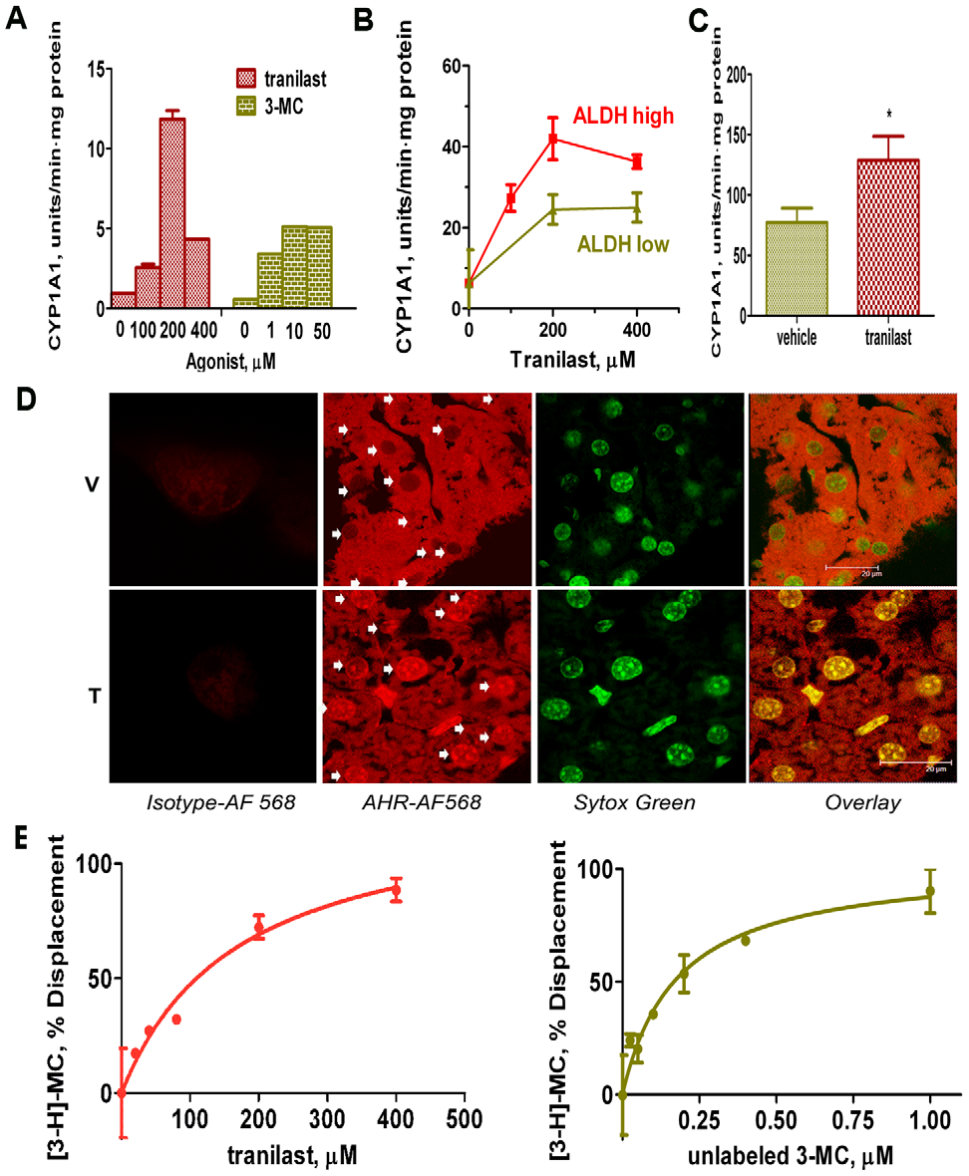
Tranilast is an AHR agonist. A. CYP1A1 induction (EROD assay) by tranilast or 3-MC (a high affinity AHR agonist). Adherent MDA-MB-231-derived D-CSCs were pre-treated for 1 h with either tranilast or 3-MC and then assayed for CYP1A1 expression by EROD assay. The enzyme activity was measured as the product-related fluorescence and expressed as arbitrary fluorescence units per min per mg protein. Tranilast induced CYP1A1 expression at higher concentrations than 3-MC, but generated higher maximum levels of the enzyme. B. ALDH high and ALDH low MDA-MB-231 cells, previously sorted by FACS, were allowed to adhere and treated as above. Higher levels of induced CYP1A1 expression were observed in the ALDH high cells. C. C57BL/6 mice were treated with tranilast twice (300 mg/kg; d 0 and d 1) and terminated on day 2 to recover liver tissue. CYP1A1 (EROD assay) was measured in liver microsomal fraction from tranilast- or vehicle-treated mice. Tranilast induced a significant increase in CYP1A1 enzyme activity (p<0.05; n = 5/group), indicating that sufficient drug levels were achieved to exert AHR agonist activity in vivo. D. Mice were treated with tranilast (T) or vehicle (V) as in Fig. 6C. Tranilast-induced nuclear translocation of AHR in vivo as detected by immunofluorescent confocal microscopy, further confirming its AHR agonist activity in vivo. Cryo-sections of liver from tranilast- or vehicle-treated C57 mice were stained against AHR (AF 568, red) and counter-stained with a nuclear-specific stain Sytox Green (green). The arrows indicate the nuclei. The images are representative of each group (n = 5/group). E. Competition of tranilast or unlabeled 3-MC with [3-H]-3-MC for binding to plate-immobilized AHR from mouse liver cytosol. The data was acquired in duplicate and is expressed as a % displacement of radioactive ligand. Ki = 44 µM for tranilast versus 0.035 µM for the non-labeled 3-MC.

As mentioned above, D-CSCs express high levels of AHR. Since Kerkvliet and colleagues suggested that tranilast is an AHR agonist [16], we hypothesized that the AHR is a major drug target in our experiments. To address this, we first confirmed that tranilast is indeed an AHR agonist. These agonists are strong inducers of the CYP1A1 enzyme [15], and we found that tranilast increases CYP1A1 expression in breast cancer cells (Fig. 6A) by EROD enzymatic assay [26] in vitro. Higher concentrations of tranilast were required for induction (Fig. 6A) as compared to 3-methylcholanthrene (3-MC), which is a classic high affinity AHR agonist [27]. Nevertheless, maximal enzyme induction was higher in the tranilast cultures, and we speculate this occurred because tranilast is less toxic than 3-MC. The level of CYP1A1 expression observed with many agonists reaches a peak and then declines at higher concentration. This might be related to toxicity or other mechanisms. CYP1A1 induction was higher in ALDH^hi^-sorted cells derived from the untreated parental cell line (Fig. 6B), consistent with a higher expression of the AHR by these cells as determined by flow cytometry (data not shown).

In vivo, tranilast significantly increased CYP1A1 expression in the liver (Fig. 6C), and induced translocation of the AHR to the nucleus as analyzed by confocal microscopy (Fig. 6D). This demonstrates that sufficient drug concentrations are achieved in vivo by our therapy to activate the AHR. Finally, we performed binding studies and showed that tranilast competes with 3-MC for binding to the AHR (Fig. 6E). These in vitro and in vivo findings are all consistent with AHR agonist activity. The affinity of tranilast for the AHR (Ki = 44 µM; calculated with the Cheng-Prussof equation) is considerably lower than 3-MC (Ki = 0.035 µM) but, nevertheless, it activates the AHR at concentrations (50–200 µM) that are achieved in the circulation of patients treated with tranilast (see Discussion).

### The AHR is the target of tranilast in cell proliferation assays

**Figure 7:**
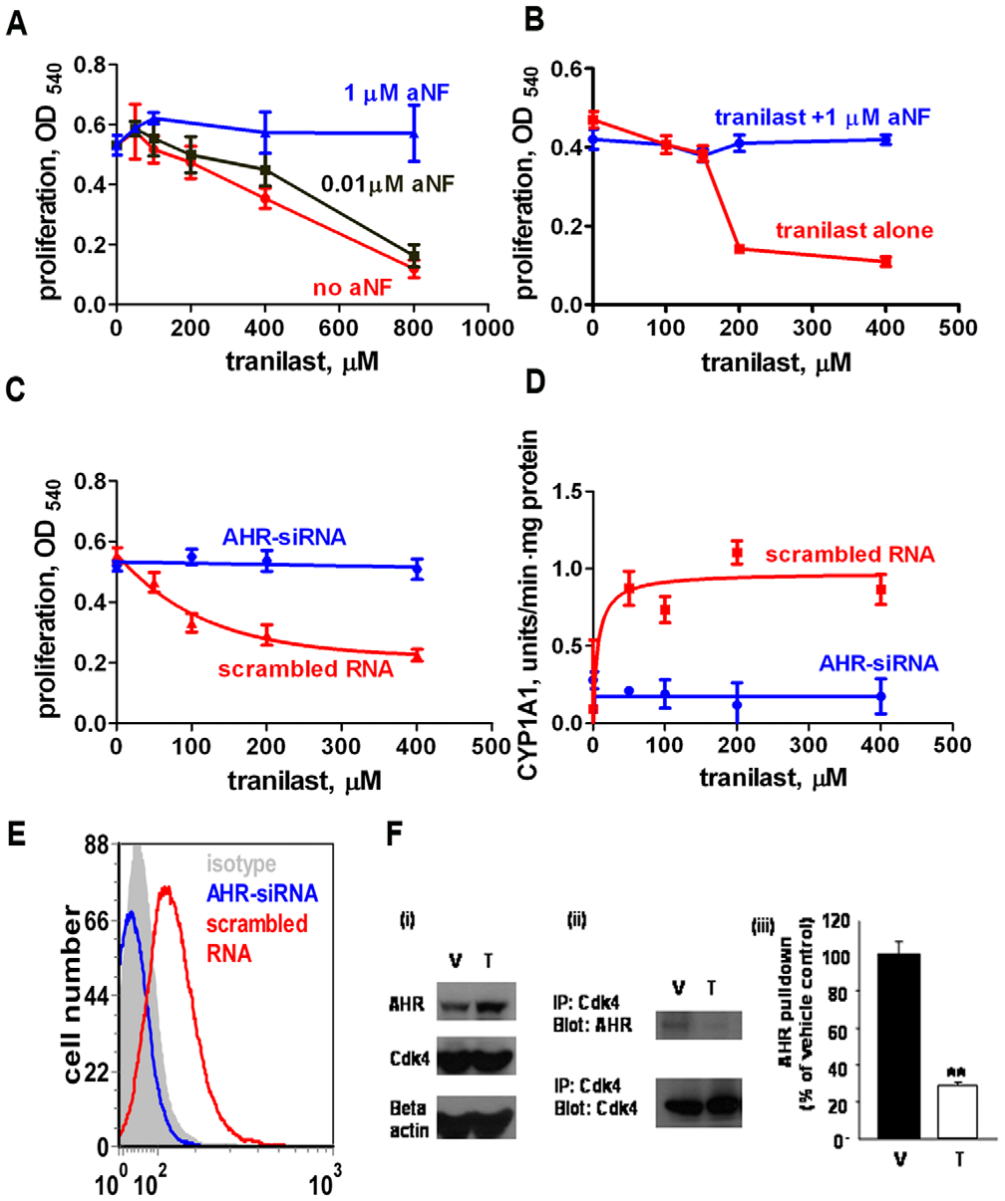
Tranilast's anti-proliferative effect is mediated through AHR stimulation. A. The suppressive activity of tranilast on proliferation of MDA-MB-231 cells was reversed by an AHR antagonist, α-naphthoflavone (aNF). Blockade of tranilast was complete at 1 µM of aNF. B. Blocking effect of aNF on BT474 cell line at a concentration of 1 µM. C. siRNA knockdown of AHR in MDA-MB-231 cells abrogated the antiproliferative effect of tranilast. D. The induction of CYP1A1 expression by tranilast in MDA-MB-231 cells was prevented by AHR knockdown. E. Transfection with AHR-targeted siRNA completely prevented AHR expression by MDA-MB-231 cells as detected by flow cytometric analysis, demonstrating the efficacy of the knockdown procedure. F. Tranilast blocked AHR binding to CDK4. Western blot analysis of lysates of MDA-MB-231 cells grown in conventional DMEM-based medium with vehicle (V) or tranilast (T) (200 µM; 48 h culture) was preformed as described under Materials and Methods. Tranilast enhanced AHR expression, but not CDK4 expression (C i). Immunoprecipitation of lysates of cells grown in vehicle (V) revealed that anti-CDK4 antibody also pulled down AHR, indicating that the two molecules bind to each other, and this binding was significantly disrupted by tranilast (C ii, iii, **p<0.001). These results are representative of 3 experiments.

We analyzed whether blockade of AHR signaling affected tranilast activity in our assays. The addition of an AHR antagonist to cultures, α-naphthoflavone (aNF) [15], [17], or knockdown of the AHR with siRNA, completely reversed the activity of tranilast against cell proliferation of the MDA-MB-231, BT474 and 4T1 cell lines (Fig. 7A–C). The high efficiency of the knockdown was confirmed by the loss of CYP1A1 induction by EROD assay (Fig. 7D) and loss of AHR expression by flow cytometric analysis (Fig. 7E).

### Tranilast disrupts the interaction of AHR with CDK4

Here, we found that tranilast moderately increases the expression of the AHR in MDA-MB-231 breast cancer cells (Fig. 7F,i). Furthermore, we observed in coprecipitation assays that AHR coprecipitates with CDK4, and this interaction is disrupted by tranilast (Fig. 7F, ii, iii). It is notable that although tranilast moderately increased the amount of AHR, the amount of AHR coprecipitated with CDK4 was decreased in these cells. This supports the conclusion that tranilast inhibits the binding of AHR and CDK4. Barhoover et al. [28] reported that in the absence of an external ligand the AHR interacts with CDK4, and this promotes the phosphorylation of RB, allowing the cell cycle to proceed. However, in the presence of an external AHR ligand (TCDD) the association of AHR with CDK4 was blocked, resulting in much reduced RB phosphorylation and cell cycle arrest. Our results suggest that tranilast is acting similarly to TCDD by preventing the association of AHT and CDK4, which might explain reduced RB phosphorylation (as in Fig. 2D), but further studies are required to confirm this.

### The AHR is the target of tranilast in mammosphere assays

**Figure 8:**
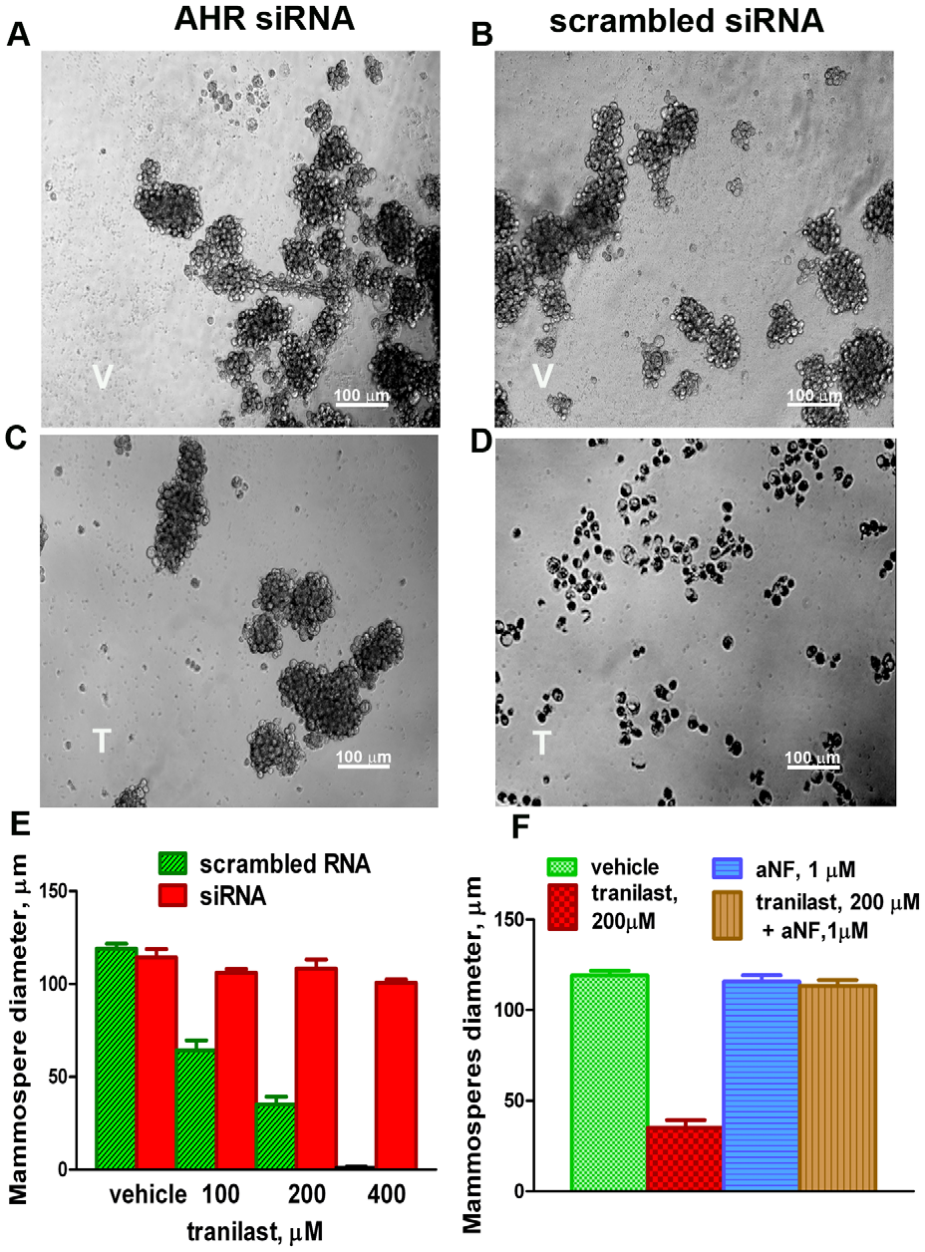
Tranilast's anti-mammosphere effect is mediated through AHR stimulation. A–D. Mammospheres formed by MDA-MB-231-derived D-CSCs, transfected with AHR-targeted (A, C) or scrambled siRNA (B, D) were treated with 200 µM tranilast (C, D) or with vehicle (A, B) for 48 h. T =  tranilast; V =  vehicle. Tranilast dissociated the mammospheres formed from cells expressing AHR (scrambled siRNA), but had no effect on cells with AHR knockdown (AHR siRNA), demonstrating the AHR is required for this tranilast effect. E. The diameter of mammospheres as a function of the concentration of tranilast in AHR-expressing versus knockdown cells. F. Tranilast's anti-mammosphere effect was completely blocked the AHR antagonist aNF, further confirming the requirement of the AHR. The data is representative of two independent experiments.

As with proliferation, we found that the anti-mammosphere activity of tranilast was completely abrogated by siRNA-mediated knockdown of the AHR (Fig. 8A–E) or addition of aNF to cultures (8F). Remarkably, mammospheres were completely protected from the effects of tranilast by these procedures, suggesting that the AHR is the major and possibly the sole molecular target of this drug in these assays. Note that the increase in cell death induced by tranilast in mammosphere cultures was prevented by the AHR antagonist aNF (Fig. S1B).

### Anti-cancer effect *in vivo*

**Figure 9:**
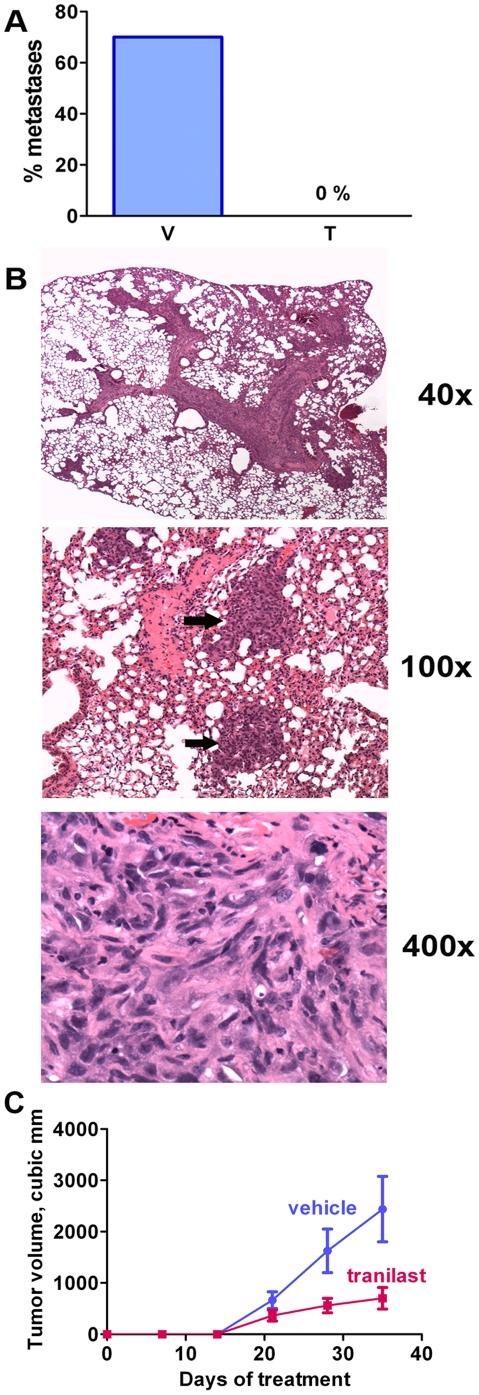
Tranilast prevents breast cancer metastasis in a xenotransplantation model. A. NOD scid gamma mice received a single i.v. injection of MDA-MB-231-derived D-CSCs (150,000 cells; day 0), and were treated with either vehicle (V) or tranilast (T; 300 mg/kg/d) from day -1 to day 21. The mice were terminated on day 28 and the lungs were examined. Lung metastases were observed in the majority of vehicle-treated mice, but there was not a single metastasis in the tranilast-treated group. n = 10 mice/group. p = 0.0015 by Fisher's exact test comparing the number of mice with metastases in each group. B. Metastases in the lungs of a vehicle-treated mouse (H & E staining of histological slide). The arrows indicate metastatic tumor foci. C. Inhibition of the growth of primary tumors in the mammary fat pad. 20,000 D-CSC cells were injected in a mammary fat pad at day 0. Mice were treated with 300 mg/kg/d tranilast or vehicle, starting at d 0. Tumors were examined as described [12] and volume  =  length X (width X width)/2, +/− SEM (n = 7; p<0.05 for tranilast versus vehicle).

We have previously shown that tranilast suppresses tumor growth and metastasis following the injection of 4T1 mammary carcinoma cells in the mammary fat pad of female BALB/c (syngeneic to the tumor) mice [12]. Here, to determine whether tranilast could be effective against human D-CSCs, we injected mitoxantrone-selected MDA-MB-231 cells i.v. into NOD scid gamma (NSG) mice to examine lung metastasis, or injected the cells into the mammary fad pad to examine primary tumor growth (Fig. 9A–C). These immunodeficient mice lack B and T lymphocytes (scid phenotype), and also lack NK cells and are deficient in innate immunity due to knockout of the IL-2Rγ chain.

Similarly to our previous results with unselected 4T1 cells [12], we found that tranilast inhibited the growth of mitoxantrone-selected D-CSCs injected into the mammary fat pad of female mice (Fig. 9C). In control mice the tumors grew rapidly and they had to be terminated, usually before developing metastases. We attempted to treat mice with aNF (with or without tranilast) to block AHR activation, but because of excessive toxicity and poor survival these experiments could not completed.

To test the effect of tranilast on metastatic spread, we used an i.v. lung metastasis assay, which tests the final steps of metastasis. MDA-MB-231 cells are not efficient at forming lung metastases following i.v. injections, but this is improved by injecting CSC-enriched subpopulations. Thus, Croker et al. [7] reported that 5×10^5^ ALDH^lo^CD44^low/−^CD24^+^ (non-CSC population) MDA-MB-231 cells injected i.v. into NOD scid gamma mice could establish themselves in the lungs, but they appeared unable to grow. However, when they injected the same number of ALDH^hi^CD44^+^CD24^−^ (CSC-enriched) cells, they observed sustained growth of metastatic cells. In our case, mitoxantrone-treated MDA-MB-231 cells were markedly enriched for cells with stem cells markers (e.g., ALDH, Oct-4, c-kit), and we found that that i.v. injection of 1.5×10^5^ of these cells into NOD scid gamma mice generated lung metastases in the majority of the mice. These were identified histologically at 4 weeks post cell injection (Fig. 9A,B). In this experiment, we treated the mice with tranilast (300 mg/kg/d) or vehicle from day -1 to day 21, and terminated the mice at day 28. In sharp contrast to the vehicle-treated mice that had metastases, the tranilast-treated mice did not have a single metastasis in the lungs (Fig. 9A) (p = 0.0015 by Fisher's exact test). Thus, tranilast completely prevented metastasis of a triple-negative cell line in this xenotransplantation study.

## Discussion

In this study, we show that tranilast has marked inhibitory activity against CSCs, and this is dependent on the AHR. Importantly, tranilast was active against previously untreated CSCs and those that had survived culture with a chemotherapeutic drug (D-CSCs). We used drug selection of CSCs because this likely duplicates, at least in part, some events that occur during chemotherapy of cancer patients. As recently reviewed [4], there have been two main approaches to isolate CSCs, i.e., candidate versus operational approaches. In the candidate approach specific markers are used to sort the cells. These approaches enrich for CSCs, but do not appear to define the entire CSC population [4]. For instance, in breast cancer, the ALDH^hi^ marker appears to identify more CSCs than the classical CD44^+^CD24^−/lo^ markers [6]. These subpopulations can be further fractionated with other markers, and there might be several subtypes of CSCs.

In operational approaches, the CSCs are isolated by taking advantage of CSC features such as sphere formation, or resistance to radiotherapy and/or chemotherapy [4], [8]–[10], [29]–[34]. Chemotherapeutic drugs have been shown to enrich for CSCs both in vitro and in vivo. Drug-based operational approaches have been successfully used to enrich CSCs in several cancer cell types (e.g., glioblastoma, breast, and colon) [8]–[10], [32]–[34], and do not represent a novel approach. Several chemotherapeutic drugs were effective in these studies, including doxorubicin, etoposide and mitoxantrone, and the effect does not appear drug specific. ABCG2^+^ side population (SP) breast cancer cells are strongly resistant to chemotherapeutic drugs, and these CSC-like cells are highly tumorigenic [33], [34]. Drug-induced enrichment of CSCs is clinically relevant, as shown in treated breast cancer patients [31]. To select CSC-like cells, we used an in vitro operational approach (drug selection) based on the existing literature. In view of this, we did not attempt to duplicate all assays reported in previous publications.

We observed that a 5-day incubation in mitoxantrone resulted in massive cell death, but 30–50% of the drug-surviving cells expressed stem cells markers such as ALDH^hi^, c-kit, Oct-4 and ABCG2. The parental cells expressed AHR, but this was also markedly increased by mitoxantrone selection. Our results are in accord with previous enrichment for MCF-7 SP cells with mitoxantrone [29], as well as the ability of ABCG2 to pump mitoxantrone out of the cells [21], [22]. Other investigators have shown that ABCG2 and c-kit are markers of drug-resistant, highly tumorigenic SP cells (CSCs) of MDA-MB-231 and MCF-7 cell lines [33]. Similarly, ALDH^hi^ is a marker for tumorigenic breast CSCs [6], [7]. Although we found that drug selection enriched for CSC-like cells, our marker studies suggest the resulting population is a mixture of CSCs and other cells. This is likely a limitation of all current breast CSC selection methods.

The mitoxantrone-selected cells were considerably more efficient at forming mammospheres. Mammospheres formation depends on CSCs, and growth of breast cancer cells in this assay results in an enrichment of Oct-4+ CSCs [35]. Furthermore, our data show that drug-surviving MDA-MB-231 cells (∼50% ALDH^hi^ cells) injected i.v. into immunodeficient NSG mice form lung metastases (see below), which is consistent with the presence of tumor-initiating CSCs. As reported by Croker et al. [7], CD44^−/lo^ALDH^lo^ (Aldefluor assay) MDA-MB-231 cells injected i.v. into NSG mice did not grow after implanting in the lungs. However, injection of CD44^+^ALDH^hi^ cells generated metastases. Other authors have also reported that ALDH^hi^ cells of various breast cancer cell lines (HCC1954, MDA-MB-453 and SUM159) have increased metastatic potential, as compared to ALDH^lo^ cells [6]. Therefore, we conclude that the mitoxantrone-selected cells we generated contain a substantial proportion of CSC-like cells.

We previously found that tranilast inhibits the proliferation of several mammary tumor cell lines including mouse 4T1, rat LA7 (a CSC-like line [36]), and human breast cancer (MDA-MB-231, MCF-7, SKBR3 and BT474) (Ref. 12 and unpublished data). Of the human cell lines, SKBR3 and BT474 (HER-2^+^ lines) and MCF-7 (ER^+^ line) were moderately more sensitive to tranilast than MDA-MB-231, but all were suppressed. In the highly metastatic 4T1 cell line, tranilast inhibited TGF-β signaling, ERK1/2 and JNK phosphorylation, and EMT [12], [13]. It also caused reduced RB phosphorylation and cell-cycle arrest [13]. In vivo [12], it markedly reduced (>50%) the growth of the primary tumor. However, its effects on metastasis were more striking, with >90% reduction of metastases to the lungs and complete prevention of metastasis to the liver.

Here, we show tranilast strongly inhibits both colony and mammosphere formation by breast cancer cell lines. Inhibition occurred at tranilast concentrations ≥100 µM, and at 400 µM no mammospheres were seen. Suppression of mammosphere formation occurred equally in mammospheres produced by CSCs from non-treated cells or mitoxantrone-selected cells. Mammosphere numbers and size were reduced. Tranilast reduced cell survival in these cultures by up to ∼50%. However, at concentrations that can be achieved pharmacologically (100–200 µM) some spheres still formed, although they were much smaller, suggesting at least partial survival of sphere-forming cells.

The phosphorylation of RB and the expression of the stem cell markers CD133 and Oct-4 were also reduced. Remarkably, when tranilast was added to mammospheres that were already formed, at concentrations ≥100 µM, they were completely dissociated within 48 hours. This was not due to cell death alone, because at least 50% of the cells were still alive following dissociation. This is consistent with our previous observations that tranilast is not cytotoxic in short-term (2–3 day) cultures of parental (unselected) cancer cells [12], although in vivo it increased apoptosis in the tumors of tranilast-treated mice [13]. The dissociation of the tumor spheres is an important finding, because it suggests that the anti-mammosphere effect is not solely due to an inhibition of proliferation. When compared to high levels of paclitaxel or etoposide, tranilast appeared more effective at reducing mammosphere numbers. Tranilast is an AHR agonist (as outlined below), and this sensitivity of CSCs to tranilast might reflect high AHR expression, since mitoxantrone-treated cells expressed higher levels of this marker. Similarly, ALDH^hi^-sorted cells (enriched for CSCs) expressed higher AHR levels than ALDH^lo^ cells, and showed higher levels of CYP1A1 induction when incubated with tranilast.

The effects of tranilast against mitoxantrone-selected D-CSCs (MDA-MB-231 cell line) were similarly apparent in vivo. Primary tumor growth in the mammary fat pad was inhibited by >50%. This is similar to our findings with 4T1 cells injected into BALB/c mice [12]. We injected these cells i.v. into severely immunodeficient NOD scid gamma mice (lacking B cells, T cells and NK cells). The vehicle-treated mice developed metastatic foci in the lungs but, in sharp contrast, the tranilast-treated mice were free of metastatic tumor. Thus, it was effective against an aggressive triple-negative, drug-selected breast cancer cell line. These results are in accord with our previous findings of an anti-metastatic effect of tranilast against 4T1 cells in BALB/c mice with normal immunity [12].

We demonstrate that tranilast is an AHR agonist. The AHR is a receptor for toxins such as TCDD (frequently called “dioxin”), polycyclic aromatic hydrocarbons (PAHs) and many other ligands [15], [16], [27]. It is a member of the basic-loop-helix-PER-ARNT-SIM (PAS) family of proteins and functions as a ligand-activated transcription factor [15], [27]. It is normally latent (bound to Hsp90/XAP2 chaperon complex) in the cytoplasm. AHR agonists release AHR from this latent complex, and in the canonical pathway it then migrates into the nucleus and binds to another transcription factor denoted aromatic hydrocarbon nuclear translocator (ARNT). The AHR/ARNT complex binds to dioxin-response elements (DREs), in the promoters and/or enhancers of hundreds of genes, and either enhances or suppresses their expression. Prototypically, there is greatly increased expression of cytochrome P450 mono-oxygenases genes (CYP1A1, 1B1 and 1A2). The toxicity of TCDD is dependent on AHR expression, but the reason for its high toxicity is not well understood, and many AHR ligands are not notably toxic.

Supporting the activity of tranilast as an AHR agonist we find that: 1) It strongly induces CYP1A1 expression (a classic marker of AHR activity) in breast cancer cells. In vivo, in the liver, tranilast administration induced translocation of the AHR into the nucleus and increased expression of CYP1A1. 2) Addition of an AHR antagonist, aNF, to cultures completely reversed the activity of tranilast against cell proliferation and mammosphere formation. Tranilast no longer dissociated formed primary or secondary mammospheres, and protection was complete. 3) Knockdown of the AHR with siRNA in MDA-MB-231 abolished the activity of tranilast in the same assays as aNF. The finding that AHR knockdown prevented the anti-mammosphere activity of tranilast, such that mammospheres formed normally, strongly supports the conclusion the AHR is the primary drug target for inhibition in this assay. 4) Tranilast competed with 3-MC for binding to the AHR in receptor binding assays. Although tranilast has a much lower affinity for the AHR than 3-MC, it can induce comparable expression of CYP1A1 in breast cancer cells when added to cultures at pharmacological concentrations (≥100 µM).

The role of the AHR in toxicology has been studied for decades, but it is now clear that it has multiple other physiological functions. Indeed, gene activation through DREs does not readily explain all the effects of tranilast. However, the AHR can bind to other transcription factors, and in non-canonical pathways [28] exerts activities which are compatible with the effects of tranilast and relevant to breast cancer therapy. As reported by Barhoover et al. [28], the AHR (without an exogenous ligand) contributes to cell cycle progression by binding to CDK4 and promoting phosphorylation of RB, but this interaction is disrupted by a ligand (TCDD) resulting in cell cycle arrest [28]. Thus, in this model, the AHR without ligand facilitates cell cycle progression, whereas with an external ligand it is inhibitory. Our data confirms that tranilast also prevents the association of the AHR with CDK4, suggesting this is a mechanism for cell cycle arrest. In accord with this, tranilast greatly reduced RB phosphorylation in mammosphere assays (this study), and in breast cancer cells grown under conventional conditions [13]. We speculate that a block of AHR/CDK4 binding explains the effects of tranilast in reducing RB phosphorylation and blocking the cell cycle, based on the literature [28], but further studies are required to establish this. Other mechanisms might also contribute, because the AHR can also bind directly to the RB protein in a ligand-dependent way [37].

Interestingly, the AHR antagonizes TGF-β activity, and represses latent TGF-β binding protein-1 (LTBP-1) [38]. LTBP-1 is required for TGF-β secretion and anchors the latent cytokine to extracellular matrix components. In accord with this, AHR ligands suppress TGF-β secretion [38] and the cells of AHR-null mice produce more TGF-β [39]. In view of this, we speculate that the inhibitory effects of tranilast on TGF-β production or action are related to AHR agonism, but this question requires further study. TGF-β appears to play a role in CSC survival or differentiation [40], and this may be relevant to our findings. Furthermore, the AHR also exerts ligand-dependent inhibitory effects on NF-κB signaling [41], which might also contribute to CSC inhibition [42].

There have been few studies of AHR function in stem cells, whether normal or CSCs, and its role is unclear. AHR agonists, such as tranilast, might suppress CSCs by inducing cell-cycle arrest, or inhibiting production of growth factors and cytokines. Studies of hematopoietic stem cells (HSCs) suggest that the AHR regulates the proliferation and senescence of these cells [43]. This appears to be a normal physiological function, but in the presence of TCDD there is impaired self-renewal and depletion of HSCs. This might result from unphysiological prolonged stimulation, as TCDD has a very high affinity for the receptor and is not degraded, persisting in tissues for years. TCDD is also profoundly immunosuppressive, through actions on the thymus, peripheral lymphocytes, dendritic cells and other immune cells (16).

Interestingly, the AHR appears to be an important regulator of prostatic stem cells [44], and it protects against prostate carcinogenesis in TRAMP mice [45]. TCDD also impairs mammary tissue differentiation in mice and protects against DMBA-induced mammary tumors [46]. In some studies, contrary to expectations, exposure to TCDD and various polychlorinated hydrocarbons was associated with a reduced incidence of prostate and breast cancer [47], possibly through anti-hormonal effects although mechanisms have not been elucidated. In this respect, it is important to note that TCDD can induce degradation of the ERα through E3 ubiquitin ligase activity and exert anti-estrogenic effects [15]. This suggests that AHR agonists (including some environmental contaminants) have anti-cancer effects against some types of tumors but, as a note of caution, they have also been shown to exert carcinogenic effects. It appears that TCDD can either enhance or suppress the incidence of cancer depending on the target cell and other factors [47]. The literature on AHR agonists reveals that they do not all have the same biological actions or toxicity, for reasons that are not well understood, and some appear to be non-toxic endogenous ligands [15], [27]. Because of the promiscuous nature of the AHR, many agonists also target other molecules, which complicates the interpretation of studies. It is also unlikely that all agonists can duplicate the many activities of TCDD. Indeed, weaker agonists such as tranilast may have effects that are considerably different from those of TCDD, 3-MC and other strong agonists. Evidently, further studies are required to delineate the role of the AHR in cancer, and whether different agonists will have similar effects or not.

The biology of the AHR is complex, and activation of the AHR results in altered expression of hundreds of genes. In view of this, there are many ways in which the AHR could inhibit CSCs. The ability of tranilast and other AHR agonists to block proliferation may be important, but it does not completely explain the anti-mammosphere effect we observed. In addition to this, the AHR might act by suppressing the production of growth factors or adhesion molecules, or act by other mechanisms, and a considerable amount of future work will be required to elucidate these mechanisms.

Tranilast is of particular interest as an anti-cancer drug because of the long clinical experience in using it to treat allergy and fibrotic disease in Japan and other countries [14]. The toxicity of tranilast is remarkably low (LD50 of >1 gm/kg in rats) [48]. In human clinical studies, it is usually administered at a dose of 300 mg/day (divided in 2 or 3 doses), but in some clinical trials it has been given at doses of 600 to 900 mg/d [49]. At the higher doses ∼ 11% of patients had liver test abnormalities, which were reversible, and other side effects were relatively minor. With continuous administration of 600 mg/d, plasma concentrations of 50–200 µM have been observed [50]. In mice, absorption and pharmacokinetics appear to be different, and doses of 200 mg/kg/d (by gavage) or ∼550 mg/kg/d (added to chow) generate plasma levels in the same range as humans receiving 600 mg/d [48], [50]. We administered 300 mg/kg/d in our studies, which is a therapeutically relevant dose.

Most importantly, the AHR is expressed by ER^+^ and ER^−^ breast cancer cells, and has been found in pre-clinical studies [17], [18] to be an excellent target for the therapy of breast cancer, including triple-negative subtypes (all cell lines examined were AHR+). Indeed, AHR agonists inhibited invasive and metastatic features, and colony formation, of breast cancer cells of various phenotypes, irrespective of ER, PR or HER-2 status [17]. Our study was focused primarily on triple-negative cancer cells, because of a lack of drug targets for this breast cancer cell type, as opposed to HER2+ or ER+ cell types. However, our results with BT474 (HER2+/ER+/PR+) demonstrate that tranilast's activity is not limited to triple-negative cells. Whether all breast cancer cell types are sensitive to tranilast warrants further investigation, but studies with other AHR agonists such as TCDD [17], [18] suggest this will be the case. However, many AHR agonists are too toxic for clinical use, or have not been tested in humans, and there is a need to develop non-toxic clinically applicable alternatives such as tranilast. This is of major interest because the treatment of triple-negative tumors is problematic due a paucity of targets. Tranilast is a multi-action, non-toxic AHR agonist drug that appears effective against CSCs. It inhibits colony formation, mammosphere formation and metastasis following xenotransplantation, and may find applications in the treatment of breast cancer.

## Materials and Methods

### Mice

Immunodeficient NOD scid gamma (NSG) mice, and other mice, were obtained from Jackson Laboratory (Bar Harbor, Maine). The mice were kept in filtered cages under pathogen-free conditions. All experiments requiring animals were performed with protocols approved by St. Michael's Hospital Animal Care Committee, under approval identification number ACC940.

### Drug

Tranilast (N-[3,4-dimethoxycinnamoyl]anthranilic acid; MW = 327.3) was a kind gift of Dr. Richard Gilbert (St. Michael's Hospital, Toronto). It was dissolved in DMSO (in vitro use) or basic buffer (in vivo use), and applied as described [12].

### Flow cytometric analysis and cell sorting

Unless stated otherwise, flow cytometry analysis was performed by methods we have previously described [12], [51]. Staining and cell sorting for ALDH^hi^ cells was performed as described by Croker et al. [7], using the ALDEFLUOR™ assay kit (Stem cell technologies, Vancouver, BC). This involves an ALDH-1-dependent enzymatic reaction generating an intracellular fluorescent compound, BODIPY-aminoacetate. Its spectrum is similar to FITC. The gate to identify bright positive cells (ALDH^hi^) is set by adding an ALDH-1 inhibitor (diethylaminobenzaldehyde, DEAB) to the substrate mixture to produce a negative control. Aldefluor staining was performed as recommended by the manufacturer. The duration of the incubation with the substrate was optimized for each cell line (15–45 min at 37°C). Surface staining was performed after ALDH1 reaction, while keeping the cells in the staining buffer on ice to prevent pumping BODIPY-aminoacetate out by ABC transporters.

For cell phenotyping, we used anti-human-CD44-AF 647, anti-human CD24-PE, and anti-human c-kit (BioLegend, San-Diego, CA), biotinylated anti-human ABCG2 (eBioscience), anti-human/mouse AHR (Abcam, Cambridge, MA) and corresponding isotype controls and secondary antibodies.

### Isolation of drug-surviving CSCs

D-CSCs were generated in culture as described previously [8]–[9] with some modifications. The tumor cells were grown in medium appropriate for each cell line, supplemented with mitoxantrone (5 nM unless stated otherwise). This resulted in massive cell death; however, CSC-like cells survived and could be maintained in culture. After 5 days in drug-containing medium, the cells were grown in drug-free medium for another 3–5 days before further studies were performed. These cells formed mammospheres in culture at high frequency.

### Mammosphere culture

Mammospheres were grown in serum-free, low adherence cultures, with a CnT-27 medium and growth additives (CellnTEC Advanced cell systems, Bern, Switzerland), as described by Dontu et al. [52]. Self-renewal capacity of the CSCs was determined by producing further generations of mammospheres. At each step, the spheres were dissociated to single cells, and replated under the previous low-adherence conditions. Briefly, mammospheres were collected and centrifuged. After careful aspiration of the supernatant, 0.1% Trypsin/EDTA was added to the respective cell pellets, incubated for 1 min at 37°C. The resuspended cell pellet was then passed through a 70 µm sieve, counted and replated in mammosphere culture. In all mammosphere assays the size and numbers of mammospheres were quantitated using Image J software (NIH, USA).

### Properties of breast cancer cell lines

Studies by Neve et al. [20] and Kao et al. [53] show that breast cancer cell lines can be subdivided into similar subtypes as clinical tumors. Each cell line has a small subpopulation of ALDH-1+ cells as determined in the Aldefluor assay as previously reported [6]. The cells used in this study were obtained from ATCC, except SUM 149 and SUM159 (Asterand Inc., Detroit). ER-/PR-/HER2- (triple-negative) cell lines were MDA-MB-231 (human), SUM149 (human), SUM159 (human) and 4T1 (mouse). HER2+ cell line: BT474 (human; ER+/PR+). The cells lines were grown in DMEM, containing 10% FBS, except the SUM149 and SUM159 lines which were grown according to the supplier's instructions (Asterand Inc.).

### AHR activation assays

Functionally, expression of the AHR was detected by stimulating the cells with an agonist and detecting CYP1A1 induction with the 7-ethoxyresorufin-O-deethylase (EROD) assay. EROD assay in mouse liver microsomal fraction from tranilast-treated and control mice was performed as described by McNamee et al [26]. Final concentrations in the assay system were: 0.05 mg/ml protein, 5 µM 7-ethoxyresorufin, 2 mM NADPH, and 3 mM MgSO_4_ in PBS, pH 7.4. The reaction was performed at 37°C, started by adding NADPH and stopped after 10 min by adding a double volume of the ice-cold methanol followed by centrifugation to remove the protein pellet. The concentration of the product, resorufin, was measured at the excitation wavelength of 530 nm and emission wavelength 590 nm using Carry Eclipse spectrofluorometer.

In the case of cultured cells (MDA-MB-231), 10000 tumour cells per well in a 24-well plate were pre-treated or not with 10 µM 3-MC or various concentrations of tranilast in DMSO for 1–2 h at 37°C and rinsed with PBS. Control cells were pre-treated with the vehicle. The mix containing 5 µM 7-ethoxyresorufin and 3 mM MgSO_4_ was added to the pre-treated cells for 10–30 min at 37°C. After the incubation, the reaction mixture was aspirated and used for the measurement of resorufin accumulation as above. The cells were rinsed and used for a protein assay. Enzyme activity was expressed as an increase in fluorescence intensity (units)/min·mg protein. Assay was performed in duplicates, using normal, drug-resistant, ALDEFLUOR-sorted, and AHR-knock-down MDA-MB-231 cells.

To observe the effect of tranilast on nuclear translocation of AHR in vivo, the mice were treated with tranilast or vehicle and terminated 24 h after the last injection. Portions of fresh liver were fixed in 4% paraformaldehyde, soaked in 15 to 30% sucrose gradient for cryoprotection, and quickly frozen in O.C.T. solution on dry ice. 10 µ cryo-sections were stained with anti-AHR antibody and anti-mouse IgG-AF 568 after permeabilization and blocking and counter-stained with a nuclear dye Sytox green. The samples were studied by confocal microscopy.

### Binding of tranilast to the AHR

Binding of tranilast to the AHR was demonstrated in a competition assay using cytosolic protein from mouse liver and [3-H]-3-MC (0.2 Ci/mmol) (Moravek Biochemicals and Radiochemicals, Brea, CA). The radioligand was added at the final concentration 50 nM. The assay was performed essentially as described by Savouret et al [54]. The protein concentration was 0.85 mg/ml in HEDG buffer containing 1 mM CaCl_2_. Non-displaceable binding was assessed by adding dextran-coated charcoal (Sigma) admixed with 1% ovalbumin in HEDG buffer to the final concentration 0.15%.

In a modified assay, to minimize the contribution of cytosolic proteins other than AHR to the retention of the ligands, AHR from the cytosolic fraction was immobilized on the ELISA plate pre-coated with monoclonal anti-AHR antibody (1 µg/ml) and blocked with 2% ovalbumin. The radioactive and non-radioactive ligands (tranilast and 3-MC) pre-mixed at various ratios in HEDG buffer containing 1 mM CaCl_2_ were incubated in this plate for 2.5 h at room temperature. Unbound ligands were washed out with 0.05% TWEEN in PBS. The dried wells were separated and placed in the scintillation cocktail for counting. The assay was performed in duplicates. Both affinity-purified (antibody bound) and total cytosolic proteins had the same binding capacity for tranilast, expressed as percent displacement of the radioligand.

IC_50_ values were converted into the apparent K*_i_* value using the Cheng-Prussoff equation [55], where K*_i_* = IC_50_/[1+(L/K_D_)]. In this formula, K*_i_* is the inhibition constant for a drug (the competing ligand, i.e. tranilast or another non-labeled ligand): it represents the concentration of the competing ligand in a competition assay which would occupy 50% of the receptors if no radioligand were present. L is the concentration of free radioligand used in the assay, and K_D_ is the dissociation constant of the radioligand for the receptor. The K_i_ value for a competing ligand is an estimate of its binding determined in an independent binding or functional assay under similar conditions.

### AHR siRNA knockdown

AHR expression was determined by flow cytometric analysis and western blotting using anti-AHR antibodies from Abcam. AHR expression was knocked down with AHR-targeted SureSilencing siRNA (SA Biosciences/Qiagen). Under optimal transfection conditions using an equimolar mixture of three RNA species (A, B, and C) and SureFect transfection reagent (SA Biosciences/Qiagen), AHR expression was suppressed as determined by loss of CYP1A1 induction (Fig. 7D) and loss of AHR expression by flow cytometric analysis (Fig. 7E).

### Colony forming assay

These assays were carried out according to Korah et al, 2000 [56]. Briefly, 20,000 cells in 0.5% agar (Bacto-Agar, Difco Laboratories) were layered on preformed 0.8% agar layer using a 35 mm Petri dishes (Non tissue culture, Fisher Scientific). The cells in agar were treated with either tranilast (200 µmol/L) or vehicle (0.8% DMSO) respectively. After plating, DMEM containing 10% FBS was added to each plate. Colonies was counted under the microscope using a low magnification (4x) and photographed after 12 days.

### Western blotting

Cell lysates were prepared and Western blotting performed as described [57]. Briefly, the cells were lysed in lysis buffer (50 mM Tris pH 7.6, 150 mM NaCl, 0.1% NP-40) containing a cocktail of protease inhibitors (PMSF, leupeptin, pepstatin and aprotinin). 5X concentrated Laemmli sodium dodecyl sulfate (SDS) sample buffer containing β-mercaptoethanol was added to the cell lysates and incubated for 5 min in a boiling water bath, vortexed and an appropriate amount loaded onto a 12% SDS-PAGE for Western blot analysis.

The following antibodies were used: Rabbit anti-pRB, mouse anti-CDK4, rabbit anti-CDK4 (Santa Cruz Biotechnology, Santa Cruz, CA,), mouse anti-human β-actin (Sigma-Aldrich, St. Louis, MO,) rabbit anti-Oct-4 (Cell Signaling, Beverly, MA), rabbit anti-CD133, mouse anti-AHR (Abcam, Cambridge, MA).

### Breast cancer xenotransplantation experiments

6 weeks old female NOD scid gamma (NSG) mice (Jackson Laboratories) received a single intravenous injection (tail vein) of tumour cells, and were treated with either 300 mg/kg/d tranilast or vehicle (1% NaHCO_3_) by gavage, as we described before [12]. The physical condition of the mice was monitored daily. Metastatic disease was evaluated in lung H&E stained histological sections.

### Statistical Analysis

Statistical analyses were performed with the GraphPad Prism 3.0 program (GraphPad Software Inc., San Diego, CA). In each in vitro experiment, the significance of differences between experimental and control results was determined by either Student's t test or analysis of variance (ANOVA). Results are expressed as the mean ± SEM, unless stated otherwise. For the lung metastasis study, the difference in the number of mice developing metastases between groups was analyzed by Fisher's exact test. In all experiments p<0.05 was considered significant.

## Supporting Information

Figure S1 Replating of cells cultured with tranilast, and decreased cell survival. A. MDA-MB-231 cells were grown with 200 uM tranilast for 7 days, as described in the legend of Fig. 2A. Live cells were replated in mammosphere culture without tranilast. The figure shows that these surviving cells could still form mammospheres. B. Mitoxantrone-selected MDA-MB-231 cells were grown in mammosphere cultures with tranilast for 7 days as described in the legend of Fig. 5. The cells were recovered and examined for survival by trypan blue dye exclusion. Tranilast decreased survival in a dose dependent way, but even at the highest concentration a substantial proportion of cells survived. The AHR antagonist aNF prevented cell death.
(9.82 MB TIF)
