# Supplementary figures and images for: Breast Cancer Stem-Like Cells Are Inhibited by a Non-Toxic Aryl Hydrocarbon Receptor Agonist
Source: PLoS One. 2010 Nov 3;5(11):e13831. doi: 10.1371/journal.pone.0013831 (PMC2972222; doi:10.1371/journal.pone.0013831)

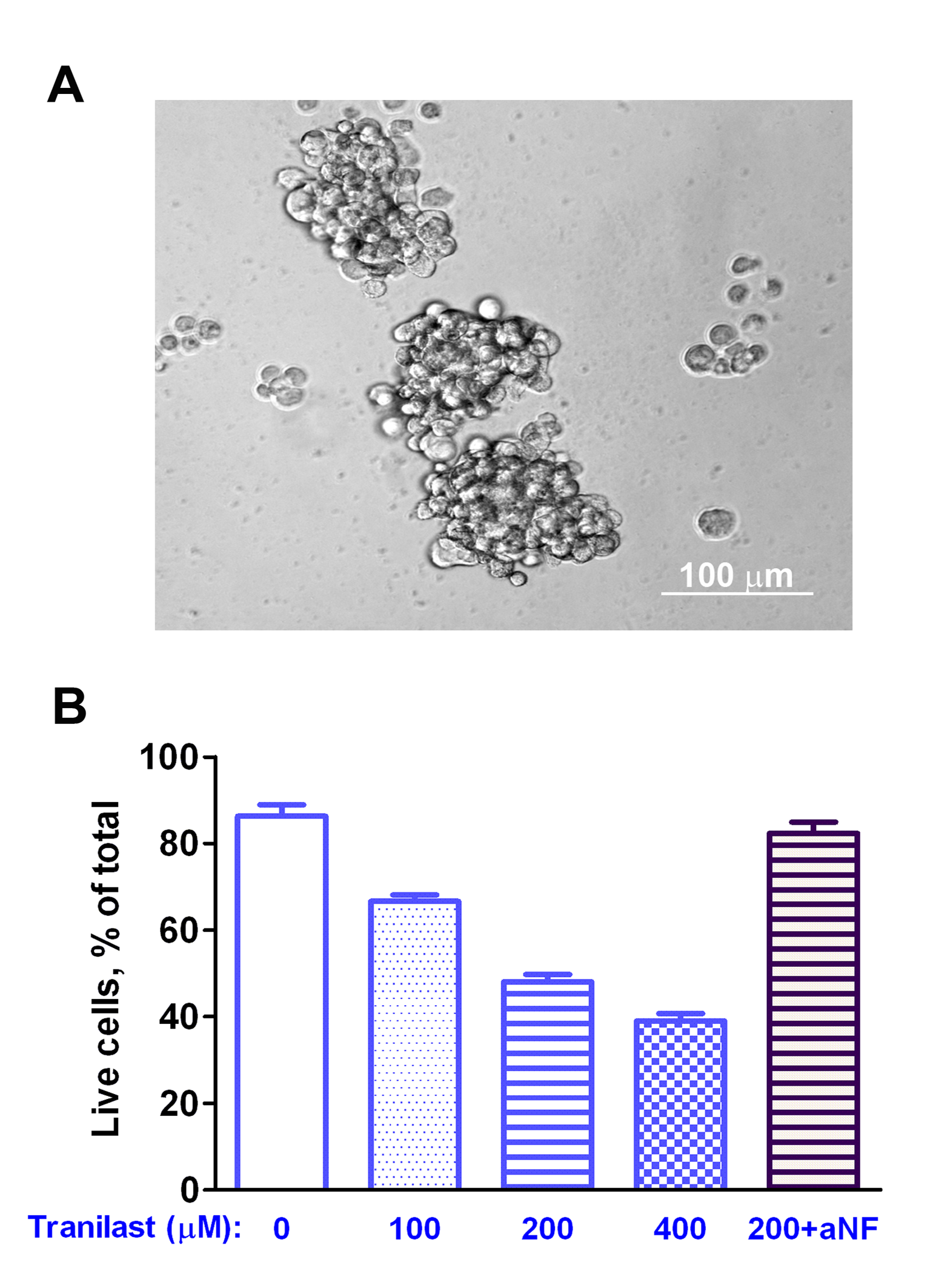

Supplement: Figure S1 — Replating of cells cultured with tranilast, and decreased cell survival. A. MDA-MB-231 cells were grown with 200 uM tranilast for 7 days, as described in the legend of Fig. 2A. Live cells were replated in mammosphere culture without tranilast. The figure shows that these surviving cells could still form mammospheres. B. Mitoxantrone-selected MDA-MB-231 cells were grown in mammosphere cultures with tranilast for 7 days as described in the legend of Fig. 5. The cells were recovered and examined for survival by trypan blue dye exclusion. Tranilast decreased survival in a dose dependent way, but even at the highest concentration a substantial proportion of cells survived. The AHR antagonist aNF prevented cell death. (9.82 MB TIF) [file pone.0013831.s001.tif]
